# Differential Gene Expression in Contrasting Common Bean Cultivars for Drought Tolerance during an Extended Dry Period

**DOI:** 10.3390/genes15070935

**Published:** 2024-07-17

**Authors:** Talita Pijus Ponce, Michely da Silva Bugança, Victória Stern da Silva, Rogério Fernandes de Souza, Vânia Moda-Cirino, Juarez Pires Tomaz

**Affiliations:** 1 Curso de Pós-Graduação em Agricultura Conservacionista, Instituto de Desenvolvimento Rural do Paraná—IAPAR-Emater, Londrina 86047-902, Brazil; 2 Laboratório de Biotecnologia Vegetal, Instituto de Desenvolvimento Rural do Paraná—IAPAR-Emater, Londrina 86047-902, Brazil; 3 Centro de Ciências Biológicas, Universidade Estadual de Londrina, Londrina 86057-970, Brazil; 4 Centro de Ciências Agrárias, Universidade Estadual de Londrina, Londrina 86057-970, Brazil; 5 Laboratório de Bioinformática, Departamento de Biologia Geral, Centro de Ciências Biológicas, Universidade Estadual de Londrina, Londrina 86057-970, Brazil

**Keywords:** *Phaseolus vulgaris*, water deficit, genetic mechanisms

## Abstract

Common beans (*Phaseolus vulgaris* L.), besides being an important source of nutrients such as iron, magnesium, and protein, are crucial for food security, especially in developing countries. Common bean cultivation areas commonly face production challenges due to drought occurrences, mainly during the reproductive period. Dry spells last approximately 20 days, enough time to compromise production. Hence, it is crucial to understand the genetic and molecular mechanisms that confer drought tolerance to improve common bean cultivars’ adaptation to drought. Sixty six RNASeq libraries, generated from tolerant and sensitive cultivars in drought time sourced from the R5 phenological stage at 0 to 20 days of water deficit were sequenced, generated over 1.5 billion reads, that aligned to 62,524 transcripts originating from a reference transcriptome, as well as 6673 transcripts obtained via de novo assembly. Differentially expressed transcripts were functionally annotated, revealing a variety of genes associated with molecular functions such as oxidoreductase and transferase activity, as well as biological processes related to stress response and signaling. The presence of regulatory genes involved in signaling cascades and transcriptional control was also highlighted, for example, LEA proteins and dehydrins associated with dehydration protection, and transcription factors such as WRKY, MYB, and NAC, which modulate plant response to water deficit. Additionally, genes related to membrane and protein protection, as well as water and ion uptake and transport, were identified, including aquaporins, RING-type E3 ubiquitin transferases, antioxidant enzymes such as GSTs and CYPs, and thioredoxins. This study highlights the complexity of plant response to water scarcity, focusing on the functional diversity of the genes involved and their participation in the biological processes essential for plant adaptation to water stress. The identification of regulatory and cell protection genes offers promising prospects for genetic improvement aiming at the production of common bean varieties more resistant to drought. These findings have the potential to drive sustainable agriculture, providing valuable insights to ensure food security in a context of climate change.

## 1. Introduction

The impact of climate change is drastically affecting global agriculture [1]. Its direct effects on production have been observed mainly due to the increase in atmospheric CO_2_ concentration, rising temperatures, and the increased occurrence of periods with water restriction. It is predicted that by the year 2100, 44% of the globe’s areas will be affected by drought, with the African continent standing out as having a higher likelihood of suffering from drought, and that production related to major crops will be halved by 2050, with over 90% losses expected by 2100 [2]. Between 1961 and 2021, climate change-related events were responsible for reducing agriculture in Africa, Latin America, and the Caribbean by 26 to 34%, suggesting that agricultural production has become more susceptible to climatic variables [3].

The Americas and Africa are, respectively, the second and third largest producers of beans globally, trailing only behind Asia. In underdeveloped or emerging countries, beans are synonymous with food security [4], being rich in iron, magnesium, zinc, potassium, and fiber, and having a high protein content [5]. They represent a viable alternative to the consumption of animal protein, which has a higher purchase value [6]. Despite its considerable importance, beans suffer drastically from water stress caused by drought [7], mainly due to their characteristic of having a shallow root system [8]. This abiotic factor threatens the food security of a wide range of regions globally [9], particularly those in Central America, South America, and Africa, which are the largest consumers of the legume [4].

Water scarcity in the soil is first detected by the roots. Recognition of the stress activates signaling pathways that will be translocated to the aerial part of the plant via xylem [10], resulting in physiological, biochemical, and molecular responses from the plant. However, the way the plant responds to the stimulus is related to the stage of development, severity, and duration of the stress [11]. During the reproductive and flowering stages, when the crop is most susceptible to drought, production losses are estimated at 58–87% due to senescence and floral abortion [12,13]. In the pod-filling stage, the reduction in productivity is lower, reaching 40% losses [14]. In addition to affecting the volume and quantity of grains, this stress is capable of altering nutritional aspects, causing a decrease in iron levels [15].

Anatomical changes in the root system such as increased cortex cell size and xylem wall thickness, decreased area of parenchymal cells, number of xylem vessels, and thickness of the epidermis and protoderm were observed in *P. vulgaris* L. subjected to water deficit [16]. In the aerial part, specifically in leaves, water restriction affects plant physiological characteristics, causing a reduction in net photosynthetic rate, stomatal conductance, respiratory rate, and chlorophyll accumulation [17]. In addition to altering the characteristics related to roots and aerial parts, the duration of the drought period affects the plant’s ability to recover from stress. Common beans are capable of resuming their development upon rehydration after 8 days of water restriction, but when kept at low water potential for 12 days, both the aerial part and root system growth are inhibited [18]. Many of the plant responses to stress are induced and/or related to the phytohormone abscisic acid (ABA). The accumulation of ABA in the roots and its subsequent distribution to the aerial part are crucial for ensuring stomatal closure, contributing to the plant’s osmotic adjustment [19,20]. Characteristics such as growth inhibition and increased root and shoot growth are attributed to higher phytohormone synthesis during dry periods [21].

Understanding plant response mechanisms to drought is fundamental when attempting to mitigate the effects caused by stress [22]. However, drought tolerance is a quantitative trait, making it difficult to manipulate in breeding programs. In this regard, some researchers aimed to discover the aspects involved in greater plant adaptability to drought by comparing tolerant and sensitive cultivars [23,24,25], highlighting the role of antioxidant enzymes such as catalase, superoxide dismutase, glutathione reductase, and ascorbate peroxidase, in plant defense. Gene expression analysis becomes crucial for understanding the metabolic pathways related to greater drought tolerance: genes such as *NAC protein*, *LEA 5*, *EF-hand calcium-binding motif*, *N3 protein*, and *S-adenosylmethionine decarboxylase* were induced by water deficit in a drought-sensitive bean cultivar, while genes related to basic metabolic processes, such as *malate dehydrogenase-like protein*, *methionine-adenosyltransferase*, and *cation:cation antiporter*, rapidly increased their expression rates in the tolerant cultivar during stress [26]. Genes related to the expression of aquaporins have been the subject of study due to the importance of these proteins in plant defense against drought [27,28]. The negative regulation of the *PvPIP2;7* and *PvTIP4;1* genes, which encode aquaporins, is involved in increased drought tolerance in beans [29], aiding in water conservation in the plant during periods of water deficit. A wide range of genes belonging to common beans, conferring resistance or susceptibility to drought, can be found in the literature [30,31,32,33,34,35]. However, the expression of these genes varies according to the cultivar, developmental stage, duration, and timing of water stress.

Given this scenario, the present study emerged with the aim of advancing the understanding of the genetic and molecular mechanisms involved in drought tolerance. Therefore, the objective was to identify differentially expressed genes in bean cultivars contrasting for drought tolerance, over a period of 20 days (the average drought period in bean-producing regions of Brazil) starting from flowering.

## 2. Material and Methods

### 2.1. Plant Material and Induction of Water Deficit in Plants

Seeds of two genotypes of *P. vulgaris* L., IAPAR 81 (tolerant) and BRS Pontal (sensitive), were used for the experiment conducted in a greenhouse located at the Institute of Rural Development of Paraná—IDR-Paraná, Londrina, PR. Phenotyping for the reaction of cultivars to water deficit was previously carried out by [25]. The experimental design used was randomized complete blocks, with split plots, with five replications. The plots were considered as stress levels: with water deficit and without water deficit, and the subplots as the cultivars (at the R5 phenological stage).

The induction of water deficit in the plants was carried out as described in [25]. The determination of substrate water retention capacity was carried out using a 30 L bucket, in which the pot was placed with 75% water relative to the volume of the bucket. After 24 h, the substrate was saturated, removed from the bucket, and when dripping ceased, its mass was measured to obtain the pot capacity. Substrate moisture monitoring was performed using a real-time gravimetric system, and water replenishment was automated according to the established water regime for each treatment. The plants were grown at 80% of pot capacity until they reached the R5 phenological stage, which, according to the bean phenological scale developed by the CIAT—International Center for Tropical Agriculture [36], is the phase in which the first floral buds emerge and notably the phase in which the plant most severely experiences the adverse effects caused by water deficit. From this moment on, the control plants continued to be cultivated with a water regime of 80% of pot capacity, while the plants subjected to drought were cultivated with 30% of pot capacity. Root collection was performed at 0, 4, 8, 12, 16, and 20 days after the onset of stress and immediately frozen in liquid nitrogen. For root collection, the substrate was removed using a brush, as the roots could not be washed since contact with water would activate rehydration mechanisms that could interfere with the expression levels of genes responsive to water deficit. Subsequently, the materials were macerated in the laboratory using sterilized porcelain crucibles and liquid nitrogen, and stored at −80 °C for subsequent RNA extraction.

### 2.2. Isolation and Analysis of Total RNA and RNASeq

The extraction of total RNA was performed according to the hot acid phenol method protocol [37], with modifications. For sequencing, 3 replicates of each treatment, with a standardized amount of 5 µg of RNA per sample, were sent for RNA-Seq using the HiSeq 2500 next-generation sequencer (Illumina, San Diego, CA, USA) at the State University of Campinas, at the Central Laboratory for High Performance Technologies in Life Sciences (LaCTAD, Campinas, Brazil), resulting in a total of 66 sequenced libraries. To ensure high-quality sequencing, the requirement was followed that the RNA Integrity Number (RIN), measured on the Agilent 2100 Bioanalyzer, was equal to or greater than 8. The cDNA libraries were constructed using the Illumina^®^ TruSeq Stranded mRNA Library Prep kit according to the manufacturer’s instructions. Sequencing was performed on the Illumina^®^ HiSeq 2500 system with single-end reads of 101 base pairs.

### 2.3. Analysis and Annotation of the Sequences

The quality of the libraries obtained from sequencing was analyzed using the FastQC v0.11.7 tool [38]. Subsequently, using TrimGalore! v0.6.4 (https://www.bioinformatics.babraham.ac.uk/projects/trim_galore/, accessed on 19 March 2024), adapter sequences were removed, as well as reads shorter than 25 base pairs and with a quality score lower than 20 on the Phred scale. Trinity v2.8.6 software [39] was used for de novo assembly of the transcriptome. To assess assembly quality, the transcriptomes were evaluated using the BUSCO v.5.3.2 database (https://busco-data.ezlab.org/v4/data/lineages/, accessed on 19 March 2024), compared with the fabales_odb10 datasets. For subsequent analyses, the largest isoform of each assembled gene that was equal to or greater than 300 nucleotides in size was chosen. Transcripts from the de novo assembly, as well as transcripts from a bean reference transcriptome [40] were annotated, via BLASTx, with a cutoff e-value of 10^−3^, against a library of 236,809 *P. vulgaris* proteins from UNIPROT (https://www.uniprot.org, accessed on 7 May 2024). For subsequent analyses, transcripts associated with proteins unique to the de novo assembly were added to the transcripts identified in the reference transcriptome. Differential expression analysis was performed with DESeq2 package v1.26.0 [41] implemented in the RStudio v2023.03.0 (https://posit.co/download/rstudio-desktop/, accessed on 7 May 2024) program in the R language. Only genes with Log_2_ fold change > 2 and padj < 0.05 were considered differentially expressed in this study and visualized through a Venn diagram [42].

Gene Ontology (GO) enrichment analysis and Kyoto Encyclopedia of Genes and Genomes (KEGG) pathway analyses were performed using g:Profiler (version e111_eg58_p18_30541362) with g:SCS multiple testing correction method applying significance threshold of 0.05 [43]. GO comprises three parts: biological processes (BP), molecular functions (MF), and cellular components (CC). Statistical significance was set at Log_2_ fold change > 2 and padj < 0.05.

### 2.4. Validation of Genes via RT-qPCR and Statistical Analyses

The cDNA synthesis was conducted using the commercial product SuperScript^®^ II Reverse Transcriptase (Invitrogen™, Carlsbad, CA, USA). For the reaction with a final volume of 20 µL, 1 µL of Oligo(dT) (0.5 µg.µL^−1^), 1 µL of dNTPs (2.5 mM each), 4 µL of 5× buffer, 1 µL of DTT (0.1 mM), 1 µL of RNaseOut (40 U.µL^−1^), 1 µL of SuperScript III RT (200 U.µL^−1^), and 11 µL of RNA were used. The concentration of the 66 cDNA samples was measured using Nanodrop 1000 (Thermo Fisher Scientific, Waltham, MA, USA) and standardized to 40 ng.µL^−1^.

The primers for RT-qPCR were designed following these criteria: (1) Tm: 60 °C, (2) amplicon size of up to 150 bp, (3) primers of approximately 20 nucleotides, (4) reaction efficiency of approximately 90–110%, and (5) whenever possible, at least one of the primers would be designed at the exon–exon junction to avoid genomic DNA contamination (Supplementary Table S1). The primer efficiency was checked by analyzing the dissociation curve performance after 40 cycles in the RT-qPCR machine, those falling between 90% and 110% being considered efficient. The RT-qPCR reactions were performed in 96-well plates using the ABI PRISM 7500 FAST sequence detection system (Applied Biosystems, Carlsbad, CA, USA), with SYBR Green as the detector. Universal cycling conditions were used (20 s at 50 °C, 10 min at 95 °C, followed by 40 cycles: 15 s at 95 °C and 1 min at 60 °C). For each gene and negative controls (blanks), technical duplicates were performed. The initial reactions contained: 6.5 µL of SYBR Green Master Mix (Life Technologies, Carlsbad, CA, USA), 0.5 µL each of forward and reverse primers, 2 µL of cDNA, and 4 µL of Milli-Q water, totaling 13 µL per reaction. The negative controls lacked cDNA and were replaced with Milli-Q water. Dissociation curves were generated after each reaction by heating from 60 °C to 95 °C at a rate of 1 °C per minute.

The expression level of each gene was quantified relative to the untreated control of each treatment within each cultivar and also between treatments of the two cultivars, using the normalizing gene T-297 [44], and demonstrated as follows: RQ = 2^−ΔΔCt^, where ΔCT = Ct (target gene) − Ct (endogenous control) and ΔΔCt = ΔCt (treated) − ΔCt (control). The statistical analyses were conducted using the Sisvar 5.7 software [45]. The data were transformed to Log_2_ to ensure greater normality. Subsequently, they were subjected to analysis of variance (ANOVA) to detect differences between treatments, where *p* < 0.05. When significant, the Student’s *t* test was applied to compare the means of the treatments within each cultivar.

## 3. Results

### 3.1. Sequence Alignment, Functional Annotation, and Analysis of Differential Expression

The sequencing of the 66 cDNA libraries generated 1529,618,114 single-end reads of approximately 101 bp each. These exhibited good quality, with few reads being discarded during the cleaning process. On average, the de novo transcriptome assembly yielded 717,719 transcripts. Analysis of assembly quality with the Busco program, using the Fabales database, identified 92.5% of the sequences, 11.1% of which were single copies, and 81.4% were duplicates. Furthermore, 1.7% of the sequences were fragmented and 5.8% were missing. Due to the high degree of gene duplication, only 172,029 transcripts were analyzed. The Blastx alignment of the reference transcriptome resulted in the identification of 62,524 transcripts, 9485 of which were unique and 53,039 in common with the transcripts identified in the de novo assembly. In turn, de novo assembly resulted in the identification of 6671 unique transcripts (Supplementary Table S2). Therefore, a total of 69,196 transcripts were subjected to subsequent analyses.

**Figure 1:**
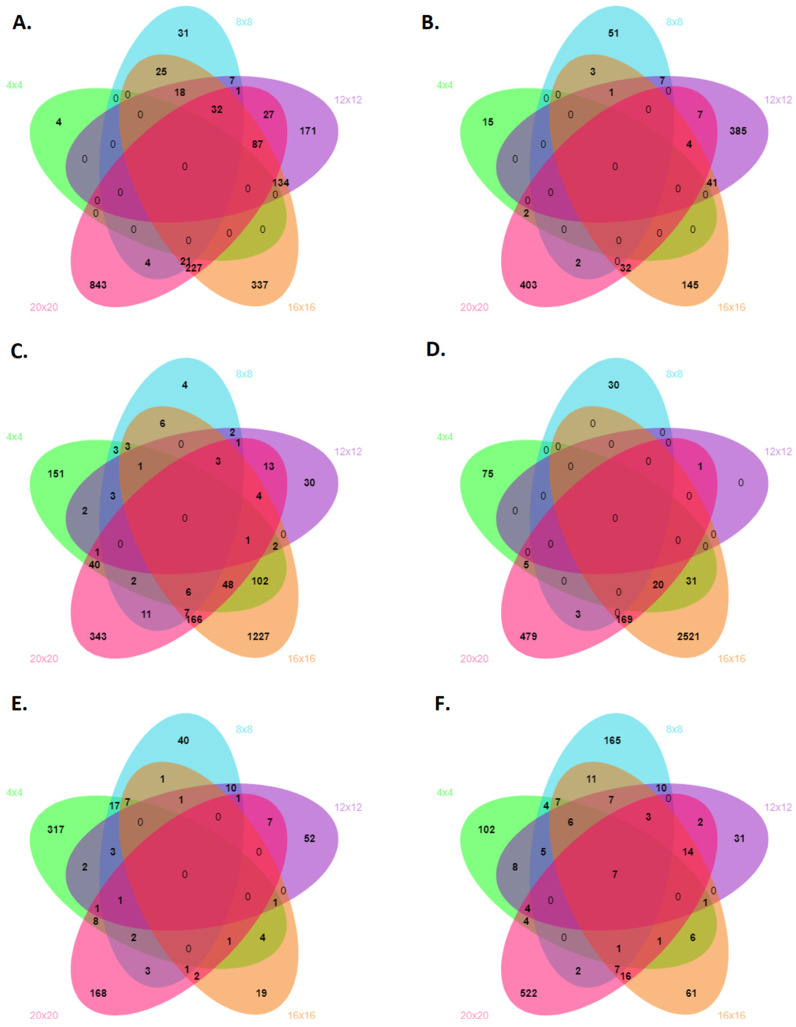
Venn diagram demonstrating the intersection DEGs at 4, 8, 12, 16, and 20 days of water deficit in common bean. (**A**) DEGs upregulated in BRS-Pontal. (**B**) DEGs downregulated in BRS-Pontal. (**C**) DEGs upregulated in IAPAR 81. (**D**) DEGs downregulated in IAPAR 81. (**E**) DEGs upregulated comparing BRS-Pontal and IAPAR 81 under water deficit. (**F**) DEGs downregulated comparing BRS-Pontal and IAPAR 81 under water deficit.

The total of differentially expressed genes (DEGs, Log_2_ fold change > 2 and padj < 0.05) in the sensitive cultivar BRS-Pontal was 20,984. The annotation by BlastX identified 8306 DEGs (7473 upregulated, and 833 downregulated by drought). At 4 days after water deficit, 173 DEGs were upregulated. DEGs downregulated were not observed at this period for the sensitive cultivar. Of the 540 DEGs at 8 days, 499 and 41 were up- and downregulated, respectively. At 12 days, 2606 DEGs, distributed as 2331 and 275 up- and downregulated, respectively. At 16 days of water deficit, 2251 DEGs were identified and, of these, 2113 and 138 were up- and downregulated, respectively. The number of regulated DEGs was higher at 20 days after the beginning of the water deficit, totaling 2736 showing changes in expression levels. Of these, 2357 and 379 were up- and downregulated, respectively (Table 1). DEGs upregulated were identified exclusively at all periods of water stress in BRS-Pontal (Figure 1A). Of the DEGs downregulated in the sensitive cultivar, 15, 51, 385, 145, and 403 were exclusively expressed at 4, 8, 12, 16, and 20 days, respectively (Figure 1B). No DEGs were observed simultaneously regulated in all periods.

For IAPAR 81, the drought tolerant cultivar, a lower number of DEGs (Log_2_ fold change > 2 and padj < 0.05) were observed, in relation to BRS-Pontal (12,441). At 4 days of water deficit, 428 and 50 DEGs were up- and downregulated, respectively, totalizing 458 DEGs observed in this period. Of the 252 DEGs identified by BlastX at 8 days, 220 and 32 were up- and downregulated, respectively. At 12 days, 619 showed changes in expression levels, and of these, 615 were upregulated and 4 were downregulated. The highest number of DEGs was observed at 16 days of water deficit: 3087. Interestingly, there were more downregulated genes (2022) than upregulated genes (1065) in this period. At 20 days of drought, 1328 DEGs were identified. Of these, 870 were upregulated, and 458 were downregulated (Table 1). The number of DEGs exclusively upregulated at 4, 8, 12, 16, and 20 days of water deficit was 151, 4, 30, 1227, and 343, respectively (Figure 1C), and the DEGs exclusively downregulated represented 75 for 4 days, 30 for 8 days, 0 for 12 days, 2521 for 16 days, and 479 for 20 days after the beginning of the drought (Figure 1D). As for BRS-Pontal, no DEGs were observed simultaneously regulated in all periods.

The comparative transcriptional analysis between tolerant (IAPAR 81) and sensitive (BRS Pontal) revealed 4679 DEGs (Log_2_ fold change > 2 and padj < 0.05). Of these, it was possible to identify 1745 genes by BlastX and 315 showed changes in expression levels at 4 days post water deficit, totaling 293 transcripts upregulated and 22 downregulated in the tolerant cultivar compared to the sensitive one. At 8 days of water deficit, 220 DEGs, totaling 62 transcripts upregulated and 158 downregulated. At 12 days of water deficit, 119 showed changes in expression levels, totaling 49 transcripts upregulated and 70 downregulated. At 16 days of water deficit, 145 showed changes in expression levels, totaling 130 transcripts upregulated and 15 downregulated. At 20 days of water deficit, 946 showed changes in expression levels, totaling 144 transcripts upregulated and 802 downregulated (Table 1). The expression of genes that showed the greatest regulation in all periods of water deficit can be observed in Supplementary Figure S1. Comparing the DEGs shared between BRS-Pontal and IAPAR 81 during the water deficit at each day of evaluation, 317, 40, 52, 19, and 168 genes were upregulated exclusively at 4, 8, 12, 16, and 20 days (Figure 1E). The DEGs downregulated in the contrast genotypes were 102, 165, 31, 61, and 522 at 4, 8, 12, 16, and 20 days, respectively (Figure 1F). Interestingly, *DUF4283 Domain-Containing Protein*, *Septum-Promoting GTP-Binding Protein 1*, *Exostosin GT47 Domain-Containing Protein*, *Calmodulin Binding Protein*, and *NB-ARC Domain-Containing Protein* were downregulated and were shared in all periods for both cultivars.

**Figure 2:**
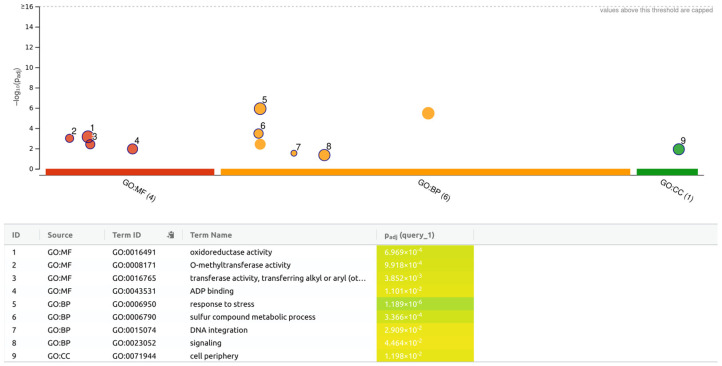
Molecular function (MF), biological processes (BP) and cellular components (CC) identified by pathways and gene ontology (GO) analyses using g:Profiler of the significant DEGs (Log_2_ fold change > 2 and padj < 0.05) when comparing BRS-Pontal and IAPAR 81 under water deficit, in all periods of evaluation (4, 8, 12, 16, and 20 days of drought). Numbers indicate the main identification GO.

The pathway and GO analyses were performed using g:Profiler with the 997 genes in the 1982 transcripts annotated (Log_2_ fold change > 2 and padj < 0.05, Table 1). The significant pathways and GO terms that were identified are shown in Figure 2. In the BP group, the selected genes were mostly associated with response to stress, sulfur compound metabolic process, and signaling. In the CC group, the selected genes exhibited a relationship with cell periphery. In the MF group, the selected genes were associated with oxidoreductase activity, O-methyltransferase activity, transferase activity, transferring alkyl to aryl, and ADP binding.

### 3.2. Water Deficit Responsive Genes

The cultivars showed different behaviors in relation to the regulation of gene expression in response to water deficiency, when comparing genes regulated in more than one evaluation period. For BRS-Pontal, sensitive to drought, the regulation of these shared genes was delayed, starting at least at 8 days after the onset of stress, as follows for *Bifunctional Inhibitor/Plant Lipid Transfer Protein/Seed Storage Helical Domain-Containing Protein*, *Non-Specific Serine/Threonine Protein Kinase*, *Cysteine-Rich Transmembrane Cystm Domain-Containing Protein*, *NAC Domain-Containing Protein 72-Like Isoform 1*, *Elongation Factor 1-Alpha*, and *ADF-H Domain-Containing Protein* (Table 2). This pattern was not observed for IAPAR 81, a drought-tolerant cultivar, in which the responsive genes began to be regulated in the initial phase of stress (4 days of water deficit). The pattern of regulation for upregulated genes is a response in the short-term, as seen for *RACK*, *TR-Type G Domain-Containing Protein*, *Elongation Factor 1-Alpha*, *Fatty Acid Hydroxylase Domain-Containing Protein*, and *Pectinesterase*, followed, at least for responsiveness, until 16 days after the beginning of the stress. For downregulated genes, such as *Glucan Endo-1*,*3-Beta-D-Glucosidase*, *Microtubule-Associated Protein 70-5*, *Peroxidase*, *Asparagine Synthetase*, and *Hexosyltransferase*, the regulation began at 4 days, with a lack of regulation until the 12th day, and downregulation again at the late periods of stress (Table 2). The comparison between BRS-Pontal and IAPAR 81 showed the drought-tolerant genes, and a distinct regulation strategy for each cultivar was observed: downregulation of genes with roles in plant defense, such as *Calmodulin Binding Protein* and *O-Methyltransferase Domain-Containing Protein*, which were regulated by water deficit in all periods of evaluation; and upregulation of genes linked to metabolism pathways, such as *Aminotransferase-Like Plant Mobile Domain-Containing Protein* and *NADP-Dependent*, with early regulation, followed by lack of regulation in some periods (Table 2).

### 3.3. Gene Expression Analysis

**Figure 3:**
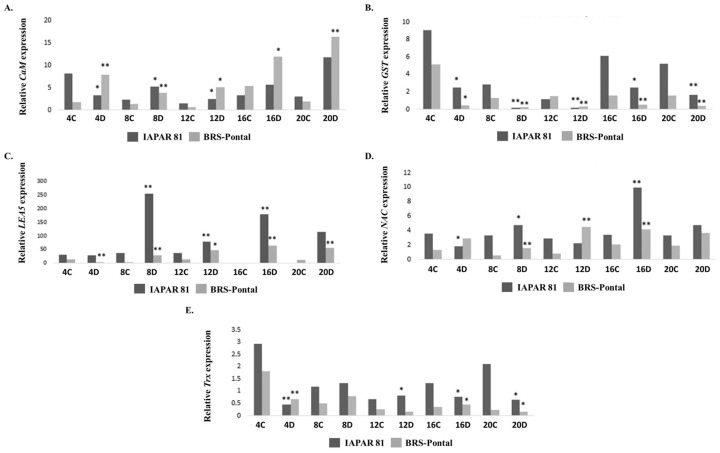
Relative gene quantification (RQ) of the *CaM* (**A**), *NAC* (**B**), *LEA5* (**C**) *GST* (**D**), and *Trx* (**E**) in cultivars IAPAR 81 (tolerant) and BRS (sensitive) demonstrated according to RQ = 2^−ΔΔCt^. Comparison between treatments subjected to drought (**D**) and controls (**C**) within each cultivar/collection period. The comparison of means was carried out using the Student’s *t* test at 5% (*) and 1% (**) of significance.

*Calmodulin* (*CaM*), *NAC, LEA5, Glutathione-S-transferase* (*GST*), and *Thioredoxin peroxidase* (*Trx*) analyzed by RT-qPCR for gene expression validation were based on the DEGs most regulated by water deficit (Supplementary Figure S1, Supplementary Table S2). In cultivar IAPAR 81, the *CaM* gene was downregulated, while at 8 and 12 days, it was induced by drought compared to its controls (Figure 3A). In contrast, in BRS Pontal, a significant induction of the gene was observed in all periods of analysis (Figure 3A). The transcriptional profile of the *NAC* gene was similar between cultivars. In IAPAR 81 and BRS Pontal, negative regulation was noted in all dry periods evaluated (Figure 3B).

Regarding the expression of the *LEA5* gene, the gene was induced at 8, 12 and 16 days in the tolerant cultivar, while in the sensitive cultivar, the gene was repressed at 4 days and induced in the remaining periods (Figure 3C). Although the cultivars present similar regulation profiles, *LEA5* regulation for IAPAR 81 was higher than for BRS Pontal. With regard to the genes that encode antioxidant enzymes, from analysis of the expression of *GST*, it was found that it was regulated from the initial period of drought in the cultivar IAPAR 81, repressed after 4 days and induced at 8 and 16 days (Figure 3D). In contrast, this gene began to be regulated only at 8 days in BRS Pontal, with induction at 8, 12 and 16 days (Figure 3D). As for *Trx*, there was repression at 4 days, induction at 12 and repression at 16 days in the tolerant cultivar under stress, while in the sensitive cultivar, repression also occurred at 4 days, induction at 16, and repression at 20 days, demonstrating that the two cultivars showed the same regulation profile (Figure 3E).

## 4. Discussion

Genes for tolerance to stresses are classified into three main categories: (1) regulatory genes involved in signaling cascades and transcriptional control; (2) genes that promote the protection of membranes and proteins and uptake/transport of water and ions and (3) genes of unknown function [46]. The first class comprises regulatory proteins that control the activities of the main enzymes of stress signal transduction pathways or modulate the expression of stress-sensitive genes. These include transcriptional regulatory factors such as DREB, AREB, MYC, MYB, bZIP, NAC, zinc fingers, ERF, and WRKY; protein kinases such as mitogen-activated (MAP), calcium-dependent (CDPKs), receptor and histidine kinases, and those related to SNF1 (SnRK2—sucrose non-fermenting-1-related protein kinase 2); phosphatases, such as the pp2c family; and enzymes involved in phospholipid metabolism such as PLD, PLC and other signaling molecules. These last two classes of proteins, together with putative unassigned proteins, work in coordination or can act independently to mitigate the effects of water deficit in plants [46,47]. The second group consists of functional proteins that include heat shock proteins, chaperones, osmotin, late embryogenesis abundant (LEA) proteins, antifreeze proteins, and mRNA binding proteins; key enzymes for the biosynthesis of osmolytes, such as mannitol, proline, betaine, glycine, and trehalose; water channel proteins and transporters, aquaporins; detoxification enzymes such as catalases, peroxidases and various proteases [46,47].

### 4.1. Regulatory Genes Involved in Signaling Cascades and Transcriptional Control

#### 4.1.1. Phytohormone Signaling Pathways

Abscisic acid (ABA) is an essential phytohormone that plays a crucial role in protecting plants against stresses, whether of biotic or abiotic origin. A vital component of this protective mechanism is the family of proteins called LEA (late embryogenesis abundant). In plants, LEA proteins are expressed in embryos in vegetative organs under water stress conditions and the final stage of embryogenesis, meaning that these proteins play a role in water deficit [48]. Studies have shown that some LEA proteins are involved in ABA signaling and protect cells against stress, especially osmotic stress [49]. The overexpression of rice *OsLEA5* confers tolerance to drought and high salinity [50], and LEA3 [51,52] and RAB16D [53] are involved in the adaptive response to water in water deficit conditions. These proteins, as observed by the transcripts identified in this study as PHASIBEAM10F023328T1; PHASIBEAM10F012908T1; PHASIBEAM10F009857T4 (induced at 4 days), PHASIBEAM10F018415T1 (repressed at 8 days) and PHASIBEAM10F003900T1 (repressed at 16 days), play an essential role in safeguarding plant cells against the effects of abiotic stress, while contributing to growth and normal plant development [48]. The significant induction, especially in the tolerant genotype, is due to the involvement of LEA proteins in protective mechanisms against environmental stresses, especially drought and dehydration [54,55]. LEA transcripts accumulate during periods of desiccation, osmotic stress, water deficit and during increased ABA levels in the plant [56,57,58]. Induction of the *LEA5* gene under water deficit is reported for the common bean [26,59], although divergences in the gene’s expression profile were found when its expression in Arabidopsis was analyzed, which was repressed [26]. Several genes encoding LEA proteins are to be found in the transcriptomic libraries of plants subjected to drought [26,60], and a progressive increase in the number of their transcripts is expected over time; however, oscillations in the regulation of *LEA5* over the evaluation time were reported [26].

The WHy (water stress and hypersensitive response domain-containing protein) domain is normally found as a component of LEA proteins that are linked to resistance to multiple stresses in different genera, and was so named because of its appearance in proteins expressed during water deficit response. Several protein families, particularly the HinI, LEA8 and LEA14 proteins, contain a unique domain, the WHy domain [61]. It was observed that hydrophilins, LEA proteins and dehydrins (PHASIBEAM10F014756T1 induced at 4 days and repressed at 20 days) confer protection against water deficit, maybe through identical mechanisms. These proteins bind to structures of the cell and reduce inactivation and denaturation, protecting from the stress by directly binding to protein surfaces and coordinated water replacement or by ordering water molecules around the associated macromolecules [62].

#### 4.1.2. Signaling and Signal Transduction

Stomatal closure is a key plant response to drought stress, controlled by guard cell turgor. ABA regulates stomatal movement, affecting ion channels, mainly inhibiting the K^+^ channel and activating the anion channel. Changes in cytosolic calcium concentration are the main transducer of ABA signaling, which can induce stomatal closure [63,64]. *CaM* and *Cam-Like* (*CML*) genes are the main families of calcium (Ca^2+^) sensors that are involved in the response to various stresses. *CaM* genes are significantly induced under drought stress in maize [65]. In alfalfa, *MsCML46* was highly induced under water deficit, cold, ABA treatments, and salt. Its overexpression increased tolerance to salt, cold, and water deficit [66].

In this study, we observed several *CaM* genes induced in almost all periods of water deficit in the tolerant cultivar (repressed at 4 days: PHASIBEAM10F027827T5; 12 days: PHASIBEAM10F027827T6; PHASIBEAM10F027826T9; 16 days: PHASIBEAM10F027826T6; PHASIBEAM10F027827T1 and 20 days: PHASIBEAM10F027826T7), and thus we can conclude that stomatal closure is a mechanism that prevents plant water loss as a long-term developmental adaptation during drought stress. *CaM* genes are among the main families responsive to stressful stimuli (cold, drought, metals and salt). Although the CaM domains are conserved, their expression is still unclear in maize [66]. *CaM* repression after 8 days of drought in tolerant and sensitive common bean genotypes was reported, although the regulation of the latter genotype was more pronounced [26]. In the present study, a greater intensity of regulation was also observed in the sensitive cultivar; however, the repression observed by [26] was observed only 4 days after the start of water deficit in the tolerant cultivar. In banana, a CaM-binding transcription factor was upregulated under drought stress and may be a candidate gene for drought tolerance [67].

#### 4.1.3. Transcription Regulatory Factors

During the gene regulation process, transcription factors (TF) play an important role, as they respond to stress signals and so control gene expression. Several families of TFs, such as zinc finger, MYB, WRKY, and NAC are involved in regulating plant resistance to drought stress [68,69]. Stress-associated proteins (SAPs), a recently identified class of zinc fingers proteins (ZFPs) (induced at 20 days: PHASIBEAM10F025156T1 and repressed at 16 days: TRINITY DN155254 C0 G1 I1), have been reported as important factors in the regulation response of multiple biotic and abiotic stresses [70]. Overexpression of *SAP* genes can significantly increase a plant’s resistance to high temperature, cold, salinity, heavy metals, and water deficit [71,72].

WRKY transcription factors constitute one of the largest families of transcription factors in plants, presenting a significant role in plant defense against water deficit. *OsWRKY45* cloned from rice was overexpressed in Arabidopsis, which increased disease resistance to pathogens and water deficit tolerance [73], indicating that *OsWRKY45* is involved in signal transduction in response to biotic and abiotic stress [74]. The *ZmWRKY58* gene isolated from corn was overexpressed in rice, which increased the tolerance of transgenic rice to salinity and water deficit [75]. The overexpression of MuWRKY3 TFs in groundnut (*Arachis hypogaea* L.) presented elevated tolerance to water deficit, indicating that MuWRKY3 TFs (nuclearly localized) controlled the expression of dehydration-response genes [76,77]. In our study, we observed the expression of WRKY TFs repressed at 8 days: PHASIBEAM10F010716T1; PHASIBEAM10F010716T2, suggesting that the expression of these genes can promote stomatal motility, water retention, and present lower reactive oxygen species (ROS) [78].

When plants are submitted to water deficit, MYB TFs regulate the expression of metabolite genes such as wax, flavonoids, and cutin, the main components of the cuticle (which protects the plants’ aerial organs from desiccation) to modulate water-deficit tolerance [79]. MYB TFs participate in stomatal movement, through ABA signaling [80]. In Arabidopsis, MYBs TFs regulate cutin synthesis genes and wax synthesis genes [81]. In this study, we observed the expression of MYB TFs (induced at 8 days: PHASIBEAM10F019042T4; PHASIBEAM10F019042T7 and 12 days: PHASIBEAM10F019042T7; PHASIBEAM10F019042T1), thus we assume that the expression of these genes in short and long periods of water deficit can modify morphological characteristics that confer the ability to save water in stressed tissues [82], and higher root vitality [83].

Since the discovery of NAC TFs in *Arabidopsis thaliana* [84], they have been reported in soybean, rice, and other plant species [85,86]. NAC TFs make up a multigene family of transcription factors, involved in plant development and responses to biotic and abiotic stresses [87,88,89]. It is suggested that the role of TF NAC in the coordination and integration of signals involves hormones, enzymes, development, programmed cell death and mainly transcriptional regulation. These genes are related to budding, root development, floral morphogenesis, hormonal signaling, leaf senescence, cell cycle control, embryonic development, and defense responses [90]. Furthermore, studies have demonstrated that the NAC family of TFs are involved in responses to hormonal signal transduction, abiotic and biotic stress, and cell apoptosis [91,92]. Overexpression of *GmSNAC49* in Arabidopsis improved water deficit tolerance by upregulating drought-related genes via ABA signaling [93]. In our study, we observed the expression of NAC TF genes induced at 4 days: PHASIBEAM10F009087T2; PHASIBEAM10F001572T1; PHASIBEAM10F022136T3 and repressed at 12 days: PHASIBEAM10F023767T2 and 20 days: PHASIBEAM10F021319T2; TRINITY DN293900 C0 G1 I1), which may be related to reduced stomatal opening and stomatal density and greater peroxidase activities [92]. Although the different expression patterns found here, and the positive regulation observed previously [26], NAC is a factor that regulates the transcription of other genes, so the negative regulation of NAC observed in the two cultivars in this study is probably a strategy for regulating their drought-responsive target genes.

### 4.2. Genes That Promote the Protection of Membranes and Water and Ion Uptake/Transport Proteins

In plants, aquaporins (AQPs) (repressed at 16 days: TRINITY DN127604 C0 G1 I2) occur in multiple isoforms in the membranes of the plasmalemma and tonoplasts, resulting in the regulation of water flow into and out of cells and, ultimately, in the transfer of water through a series of cells in the leaves and roots. The influence of AQPs on water homeostasis is important, as they contribute to transpiration rate regulation, mainly under stress development due to water deficit in the soil and high atmospheric vapor pressure deficit [94,95].

Several studies have report the key roles of RING-type E3 ubiquitin transferases in distinct processes of plant development, including organ size, growth, seed germination, light signal transduction, gametogenesis, root development, and responses to abiotic stress [96,97], demonstrating that ubiquitination is involved in diverse cellular processes [98,99]. In our study, we observed the expression of RING-type E3 ubiquitin transferase repressed at 8 days: PHASIBEAM10F027011T1 and 20 days: PHASIBEAM10F027284T1; TRINITY DN289256 C0 G1 I1; TRINITY DN389780 C0 G1 I1, which may be related to ABA-mediated stomatal closure, microtubule depolymerization, and tolerance to water deficit [100].

Abiotic stress induces the differential expression of genes responsible for raffinose synthesis (RFOs) in plants [101]. The biosynthesis of RFOs begins with the activity of galactinol synthase (GolS) (induced at 4 days: PHASIBEAM10F025042T6; PHASIBEAM10F024346T1; TRINITY DN24635 C0 G1 I3 and repressed at 20 days: PHASIBEAM10F016890T9; PHASIBEAM10F006470T1; PHASIBEAM10F019731T1; TRINITY DN24635 C0 G1 I3), a GT8 family glycosyltransferase that galactosylates myo-inositol to produce galactinol [102]. RFOs perform several physiological roles and developmental functions in plants, and can contribute to desiccation tolerance, vigor and longevity [103]. Furthermore, RFOs, mainly galactinol and raffinose, are accumulated in several species in response to environmental stresses, highlighting a protective role in survival under stressful environmental conditions [104].

Glutathione S-transferases (GSTs) (induced at 4 days: PHASIBEAM10F008311T1; PHASIBEAM10F001064T1, 16 days: PHASIBEAM10F008311T1 and repressed at 20 days: PHASIBEAM10F001058T1; PHASIBEAM10F001061T1; PHASIBEAM10F001060T1) constitute a large family of enzymes that plays a diversified range of cellular roles, including participating in the elimination of ROS, providing protection to organisms against oxidative stress under stressful conditions [105]. The overexpression of this gene in tobacco improved O^2−^ scavenging under drought conditions, resulting in greater tolerance to water deficit [106]. GSTs are directly activated by specific TFs in order to regulate water deficit tolerance through directly binding to the GST and GlnRS promoters, activating gene expression [107], such as CsGSTU8, which is positively activated by the WRKY TF, CsWRKY48, increasing water deficit tolerance in Arabidopsis by enhancing ROS scavenging under dehydration [108]. The family of GSTs is multifunctional and is involved in primary and secondary stress metabolism [108]. Our data corroborate those observed by [109], who observed negative regulation of GST between 1 and 3 days of drought, and strong induction 5 to 7 days after the onset of stress. The authors also observed an increase in expression in plants treated with exogenous proline, demonstrating a relationship between the two.

Cytochrome P450s (CYPs) (induced at 4 days: PHASIBEAM10F011005T1; TRINITY DN16309 C0 G2 I2; PHASIBEAM10F026343T1; 8 days: TRINITY DN382597 C0 G1 I1; PHASIBEAM10F000661T1, 16 days: PHASIBEAM10F026343T1; TRINITY DN1738 C1 G1 I6; PHASIBEAM10F015442T1 and 20 days: PHASIBEAM10F008837T1; PHASIBEAM10F026923T1) play diverse roles in biochemistry synthesis of hormones, sterols, fatty acids, biopolymers, cell wall components, and various defense compounds [110]. CYPs are also involved in plant protection against adverse abiotic conditions by increasing the activity of flavonoids and thus increasing antioxidant activity [111,112], as CYPs genes may be differentially expressed in response to water deficit [113].

Thioredoxins (Trxs) are disulfide reductases and, like glutathiones, demonstrate an involvement with oxidative stress and signaling for response and defense pathways [114,115]. These enzymes act to protect against reactive oxygen species (ROS), which rise under stressful conditions and trigger cellular disturbances, such as membrane destabilization, protein inactivation, DNA cleavage and even inhibition of photosynthesis [26,116]. Trxs play a crucial role in protecting plant cells against damage caused by water stress, reducing the accumulation of reactive oxygen species (ROS) and activating antioxidant enzymes such as peroxiredoxin. Furthermore, they regulate stress response proteins, such as heat shock proteins (induced at 4 days: PHASIBEAM10F010549T1 and repressed at 16 days: TRINITY DN4203 C0 G1 I3 and 20 days: TRINITY DN2607 C0 G2 I2) and dehydration proteins, preserving the structure and function of these proteins. During water stress, thioredoxins restore the redox balance of cells, ensuring that they remain functional to face stress [117]. The Trx superfamily consists of three main subclasses of oxidoreductases: Trxs, glutaredoxins (Grx) (induced at 20 days: PHASIBEAM10F006146T1; PHASIBEAM10F018660T2; PHASIBEAM10F018660T1), and protein disulfide isomerases (PDI). Trx is capable of directly reducing glutathione peroxidase (GPx), reactivating the enzyme after it has participated in the reduction in hydrogen peroxide (H_2_O_2_). This allows GPx to continue to perform its antioxidant function. This regeneration is essential to maintain GPx activity in a continuous cycle of ROS scavenging [118].

*Trxs* (repressed at 20 days: PHASIBEAM10F004585T3), which belong to the peroxiredoxin (PRX) family, are disulfide-reducing enzymes that play an important role in the response to oxidative stress and in the activation of defense pathways. Furthermore, Trxs play a crucial role in protecting against reactive oxygen species (ROS) generated during photosynthetic metabolism, particularly under stress conditions such as drought and photorespiration. They play an important role in metabolic regulation in living cells through modifications in thiol-disulfide redox exchanges [117]. It is suggested that in the temporal profile verified in the present study, specifically the repressions in the initial and final days, in both cultivars, may be one of the strategies for reducing the plant’s energy expenditure and, perhaps, investment in other genes responsive to ROS.

## 5. Conclusions

There is a complex dynamic in the gene expression of common bean lines during drought stress. A significant increase in the number of differentially expressed transcripts was observed as the stress evolved to 20 days, highlighting the adaptive response by plants to water deficit conditions. Additionally, a wide range of genes associated with specific molecular functions, such as oxidoreductase, hydrolase and transferase activities, as well as biological processes crucial to the stress response, including metabolic, cellular and biosynthetic regulation. Also noteworthy is the presence of regulatory genes involved in signaling cascades and transcriptional control, such as LEA proteins and transcription factors WRKY, MYB and NAC, which play a fundamental role in modulating the response of plants to water stress.

Furthermore, genes related to the protection of membranes and proteins, as well as the uptake and transport of water and ions, such as aquaporins, antioxidant enzymes (GSTs and CYPs) and thioredoxins were identified. These components play a crucial role in protecting plant cells against the adverse effects of water stress, regulating the stress response and contributing to the reduction in reactive oxygen species (ROS) accumulation, ensuring cellular functionality during water scarcity conditions. Finally, relative gene expression analysis revealed specific regulation patterns for genes such as calmodulin, NAC and LEA5, reflecting plants’ adaptive strategies to combat water stress. Although these results highlight the complexity of plant responses to water stress and provide valuable insights for the development of genetic improvement strategies aimed at producing crops that are more resilient to water scarcity, in a context of ongoing climate change, more information about this complex regulation is needed for understanding the relationship between the plant and the environment. 

## Figures and Tables

**Table 1:** Differentially expressed genes (DEGs, Log_2_ fold change > 2 and padj < 0.05) and number of annotated DEGs by BlastX in BRS-Pontal (sensitive) and IAPAR 81 (tolerant) common bean cultivars, in 4, 8, 12, 16, and 20 days of water deficit.

Cultivar	Number of DEGs	4 Days	8 Days	12 Days	16 Days	20 Days	Total
BRS-Pontal	Annotated	21	203	922	1107	692	2945
	Upregulated	17	64	445	226	450	1202
	Downregulated	4	139	477	881	242	1743
IAPAR 81	Annotated	496	85	64	4317	1323	6285
	Upregulated	131	33	1	2741	677	3583
	Downregulated	365	52	63	1576	646	2702
BRS-Pontal vs. IAPAR 81	Annotated	520	322	177	185	778	1982
	Upregulated	156	235	98	148	583	1220
	Downregulated	364	87	79	37	195	762

**Table 2:** Differentially expressed genes (DEGs, Log_2_ fold change > 2 and padj < 0.05) shared in the time-course analysis (4, 8, 12, 16, and 20 days of water deficit) in BRS-Pontal (sensitive) and IAPAR 81 (tolerant) common bean cultivars, and the comparison between cultivars. The minus (−) sign before the fold change values indicates downregulation by water deficit.

		Regulation at (Fold Change)				
Cultivar	DEG	4 Days	8 Days	12 Days	16 Days	20 Days
BRS-Pontal	*Bifunctional Inhibitor/Plant Lipid Transfer Protein/Seed Storage Helical Domain-Containing Protein (V7CA38)*	-	−2.8	−3.0	−2.5	-
	*Peroxidase (V7BP88)*	-	-	−3.5	−3.4	−3.6
	*Non-Specific Serine/Threonine Protein Kinase (V7CDN2)*	-	4.5	3.1	4.3	3.2
	*Cysteine-Rich Transmembrane Cystm Domain-Containing Protein (V7BXH8)*	-	2.1	2.5	3.7	5.4
	*NAC Domain-Containing Protein 72-Like Isoform 1 (T2DP29)*	-	2.4	2.3	2.6	5.3
	*Elongation Factor 1-* *Alpha* *(V5N8W1)*	-	6.1	5.2	5.6	6.2
	*ADF-H Domain-Containing Protein (V7BFY8)*	-	4.0	7.2	5.5	3.4
IAPAR 81	*RACK (B7T1N8)*	6.9	5.7	5.7	4.8	-
	*TR-Type G Domain-Containing Protein (V7BXI7)*	5.4	5.6	4.9	-	-
	*Elongation Factor 1-* *Alpha* *(Fragment)(A7l3u9)*	6.3	4.1	5.2	-	-
	*Fatty Acid Hydroxylase Domain-Containing Protein (V7AP25)*	7.2	9.0	7.7	-	-
	*Pectinesterase (V7BKF6)*	4.1	3.6	-	-	3.9
	*Glucan Endo-1*,*3-Beta-D-Glucosidase (V7CVL7)*	−6.3	-	-	−8.4	−2.3
	*Microtubule-Associated Protein 70-5 (V7BRH0)*	−3.1	-	-	−2.8	−5.5
	*Peroxidase (V7BP88)*	−2.9	-	-	−5.8	−5.8
	*Asparagine Synthetase [Glutamine-Hydrolyzing] (A9XS73)*	−2.1	-	-	−3.9	−2.6
	*Hexosyltransferase (V7BA81)*	−2.1	-	-	−2.0	−2.3
BRS-Pontal vs. IAPAR 81	*DUF4005 Domain-Containing Protein (V7C9T5)*	4.9	3.6	4.2	-	2.2
	*Aminotransferase-Like Plant Mobile Domain-Containing Protein (V7C5U2)*	2.4	-	-	2.3	2.2
	*TIR Domain-Containing Protein (V7AKT3)*	4.5	7.3	-	5.8	-
	*LRGB-Like Protein (V7CJ50)*	3.4	2.0	-	3.0	-
	*NADP-Dependent Oxidoreductase Domain-Containing Protein (V7APC5)*	4.2	3.4	-	3.0	-
	*FAS1 Domain-Containing Protein (V7BYV1)*	−12.8	−7.6	−10.4	−9.2	-
	*O-Methyltransferase Domain-Containing Protein (V7C4M2)*	−3.8	−2.7	−2.3	−2.7	-
	*DUF4283 Domain-Containing Protein (V7CB55)*	−9.7	−8.2	−9.0	−7.9	−7.3
	*Calmodulin Binding Protein (Fragment) (V7BIM0)*	−6.8	−5.3	−5.3	−5.2	−5.5
	*NB-ARC Domain-Containing Protein (V7AF13)*	−4.2	−5.7	−4.9	−5.1	−3.3

## Data Availability

No new data were created or analyzed in this study. Data sharing is not applicable to this article.

## Supplementary Materials

The following supporting information can be downloaded at: https://www.mdpi.com/article/10.3390/genes15070935/s1, Figure S1: Heatmap for the differentially expressed genes (DEGs) that suffered greater regulation by water deficit between the cultivars IAPAR 81 (tolerant) and BRS-Pontal (sensitive), whose Log_2_ fold change > 2 and padj < 0.05. Red indicates negative regulation (repression) and yellow represents positive regulation (induction) of genes. (A) 4 days of water deficit; (B) 8 days of water deficit; (C) 12 days of water deficit. (D) 16 days of water deficit; (E) 20 days of water deficit.; Table S1: Sequence, annealing temperature (AT °C), and amplicons of the primers selected for Real-time qPCR validations.; Table S2: Transcripts aligned to sequences from the *de novo* assembly, using as reference the genome available in [40] for *P. vulgaris*. NCBI non-redundant protein database using the BLASTx algorithm identified in common bean cultivars contrasting for drought tolerance during 4, 8, 12, 16, and 20 days of water deficit.
