# Supplementary material for: Differential Gene Expression in Contrasting Common Bean Cultivars for Drought Tolerance during an Extended Dry Period
Source: Genes (Basel). 2024 Jul 17;15(7):935. doi: 10.3390/genes15070935 (PMC11276061; doi:10.3390/genes15070935)
Supplement: Supplementary file 1 [file genes-15-00935-s001.zip › Supplementary Figure S1.pdf]

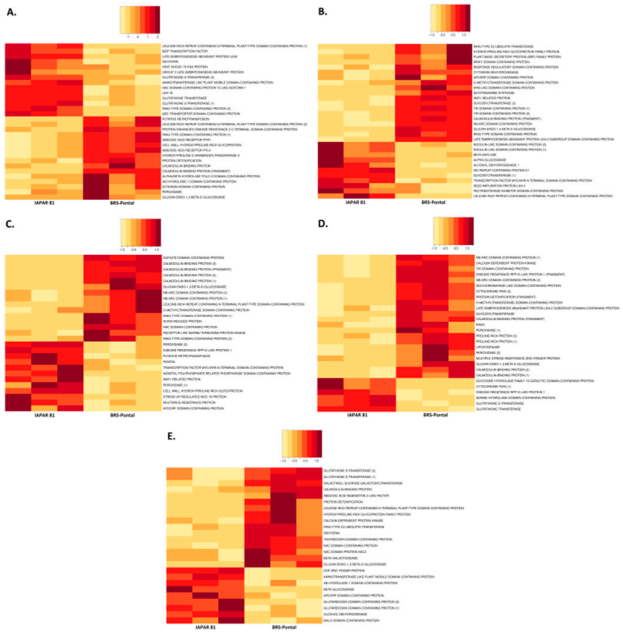

Supplementary Figure S1. *Heatmap* for the differentially expressed genes (DEGs) that suffered greater regulation by water deficit between the cultivars IAPAR 81 (tolerant) and BRS-Pontal (sensitive), whose Log2 fold change > 2 and padj < 0.05. Red indicates negative regulation (repression) and yellow represents positive regulation (induction) of genes. (**A**) 4 days of water deficit; (**B**) 8 days of water deficit; (**C**) 12 days of water deficit. (**D**) 16 days of water deficit; (**E**) 20 days of water deficit.
