# Supplementary material for: Differential Gene Expression in Contrasting Common Bean Cultivars for Drought Tolerance during an Extended Dry Period
Source: Genes (Basel). 2024 Jul 17;15(7):935. doi: 10.3390/genes15070935 (PMC11276061; doi:10.3390/genes15070935)
Supplement: Supplementary file 1 [file genes-15-00935-s001.zip › Supplementary Table S1.pdf]

Supplementary Table S1. Sequence, annealing temperature (AT ºC), and amplicons of the primers selected for Real-time qPCR validations.

Gene	Primer sequence	AT	Reference
LEA5	F: CTGTTCATCGGCGAGGTTAT<br>R: TCATGGGCCTGTAGTCACCT	62.0	P.U.
NAC domain protein	F: TGAAACCCCTGCGTAAAATC<br>R: TTCGGGGAATAATCAAGTGG	62.0	P.U.
Glutathione-S-transferase (GST)	F: AAGAAAGAGCAGGTGCATGGA<br>R: TGTTGTCGCCTCCGAAGAAT	60.0	P.U.
Calmodulin (CaM)	F: CAAGGAAACCGACCCAAGA<br>R: TCCCATCCCCATTAGAGTCAAT	60.0	P.U.
Tiorredoxina peroxidase (*Txr*)	F: CAGACGGCATTTTGGCTTAT<br>R: TCCACACCCTTTCCTTTCAG	55.0	[26]
*T197**	F: TGGGCAATTGGACGTTATTAG<br>R: GCCACGGTCTTGAACATAAAA	60.0	[41]

* Endogenous control. P.U. - Previously unpublished.
